# Enhanced *in vitro* antibacterial effect against *Enterococcus faecalis* by using both low-dose cetylpyridinium chloride and silver ions

**DOI:** 10.1186/s12903-023-02972-6

**Published:** 2023-05-17

**Authors:** Silei Lv, Wei Fan, Bing Fan

**Affiliations:** The State Key Laboratory Breeding Base of Basic Science of Stomatology (Hubei-MOST), Key Laboratory of Oral Biomedicine Ministry of Education, School and Hospital of Stomatology, Wuhan University, 237 Luoyu Road, Wuhan, 430079 China

**Keywords:** *Enterococcus faecalis*, Cetylpyridinium chloride, Silver ion, Root canal, Antibacterial agent

## Abstract

**Background:**

*Enterococcus faecalis* (*E. faecalis*) is frequently isolated from root canals with failed root canal treatments. Due to the strong ability of *E. faecalis* to resist many often-used antimicrobials, coping with *E. faecalis* infections remains a challenge. The aim of this study was to investigate the synergistic antibacterial effect of low-dose cetylpyridinium chloride (CPC) and silver ions (Ag^+^) against *E. faecalis in vitro*.

**Methods:**

The minimum inhibitory concentration (MIC), minimum bactericidal concentration (MBC) and the fractional inhibitory concentration index (FICI) were used to confirm the existence of the synergic antibacterial activity between low-dose CPC and Ag^+^. Colony-forming unit (CFU) counting, time-killing curve and dynamic growth curve were used to evaluate the antimicrobial effects of CPC and Ag^+^ combinations against planktonic *E. faecalis*. Four weeks biofilms were treated with drug-contained gels to determine the antimicrobial effect on biofilm-resident *E.faecalis*, and the integrity of *E.faecalis* and its biofilms were observed by FE-SEM. CCK-8 assays was used to test the cytotoxicity of CPC and Ag^+^ combinations on MC3T3-E1 cells.

**Results:**

The results confirmed the synergistic antibacterial effect of low-dose CPC and Ag^+^ against both planktonic and 4-week biofilm *E. faecalis*. After the addition of CPC, the sensitivity of both planktonic and biofilm-resident *E. faecalis* to Ag^+^ improved, and the combination showed good biocompatibility on MC3T3-E1 cells.

**Conclusions:**

Low-dose CPC enhanced the antibacterial ability of Ag^+^ against both planktonic and biofilm *E.faecalis* with good biocompatibility. It may be developed into a novel and potent antibacterial agent against *E.faecalis*, with low toxicity for root canal disinfection or other related medical applications.

## Background

*Enterococcus faecalis* (*E. faecalis*), a facultative anaerobic gram-positive bacterium [1] and considered to be one of main pathogens of refractory apical periodontitis [2, 3], is frequently isolated from root canals after failed root canal treatments [4, 5]. *E. faecalis* can penetrate deep into dentin tubules and form biofilms, which enhance its ability to survive nutrient deficiency and harsh environments [5, 6]. In addition, due to the complex anatomy of the root canal system, biofilms may continue to exist even after complete root canal preparation [1, 4]. Moreover, the existence of biofilms could also improve the ability of *E. faecalis* to withstand most of the endodontic disinfecting agents and antibiotics [7, 8]. As the biofilm-infection could lead to an inflammatory reaction of the periapical tissue [2], effective supplementary disinfectants are required to help control the intracanal *E. faecalis* biofilms.

Metals and their compounds have been used as inorganic antibacterial agents for a long time due to their potent antimicrobial properties [9, 10]. Currently, metal ions, especially silver ions (Ag^+^), have been regarded as a class of promising antimicrobials for dealing with the antibiotic resistance of bacteria [9]. Due to its broad-spectrum antibacterial activity, Ag^+^ has been widely used in various medical applications [11, 12]. However, Ag^+^ has high cytotoxicity and has been linked to allergic contact dermatitis, eye irritation, and irreversible discoloration after prolonged exposure [13, 14]. Furthermore, the excessive clinical application of Ag^+^ may result in Ag^+^-resistant bacteria [15]. A previous study has demonstrated the decreased susceptibility of *E. faecalis* to Ag^+^ after repeated contact [16]. Thus, it seems inadequate to use only Ag^+^ treatment for *E. faecalis* infections in root canals.

Currently, the combination of antibacterial agents which can enhance the antibacterial effect, as well as reduce the potential antibacterial resistance, is recognized as a promising strategy for controlling oral or dental infections [17–19]. Among these agents, chlorhexidine (CHX) is the most commonly used, either alone or in combination with other agents, in oral and dental disinfectants due to its notable broad-spectrum antibacterial activity [14, 20]. However, CHX may cause tissue discoloration, sensory changes, and even anaphylactic reactions and could also lead to tolerance, or even resistance and cross-resistance, to antibiotics in oral bacteria after long-term use [21, 22]. Therefore, CHX is not suitable for long-term application.

Cetylpyridinium chloride (CPC), is a cationic amphiphilic quaternary compound with excellent broad-spectrum antibacterial activity [23]. It has been used in oral hygiene products to control oral infection and supra-gingival plaque for decades [24–26]. Unlike CHX, CPC is less reported to induce resistance in *E. faecalis* [27] and has also been combined with other agents or materials to improve its antibacterial properties, as well as decrease its side effects, such as drug resistance [28, 29]. Recently, CPC has been studied for endodontic applications, and a new endodontic sealer containing high-dose CPC shows a long-term antibacterial effect against *E. faecalis* [30, 31]. However, CPC has showed high cytotoxicity at high concentrations, and is only considered safe at the concentrations under 0.1% [23], which means that the antimicrobial agent CPC is only safe at low doses, but no studies could be found at the moment about the use of low-dose CPC combined with other agent as a root canal disinfectant.

Based on these knowledge, the aim of this study was to investigate the potential of low-dose CPC + Ag^+^ as root canal disinfection against both planktonic and dentin-biofilms *E. faecalis in vitro*.

## Materials and methods

### Bacterial suspension and reagents

Strains of *E. faecalis* ATCC 29212 (ATCC, Manassas, VA, USA) were stored at -80 °C in 30% glycerol. Before testing, *E. faecalis* strains were cultured in Brain Heart Infusion (BHI, BD Biosciences, Bergen, New Jersey, USA) broth at 37 °C in an anaerobic incubator (with 5% CO_2_ and 1% O_2_). Then, the optical density (OD) of the bacterial suspension was adjusted to 1.0 at a wavelength of 600 nm (OD_600_) using a spectrophotometer (UV-2401PC, Shimadzu Corporation, Japan), which corresponds to a bacterial density of nearly 1 × 10^9^ CFU/mL [32].

All reagents were of analytical grade. CPC was purchased from Aladdin Industrial Corp. (Shanghai, China) and the Ag^+^ was derived from silver nitrate (AgNO_3_, Sinopharm Chemical Reagent Corp., Shanghai, China). All test solutions were prepared using sterile distilled deionized water (ddH_2_O).

### Minimum inhibitory concentration (MIC) and minimum bactericidal concentration (MBC) determination

The CPC and AgNO_3_ solutions were prepared using the serial two-fold dilution method [33] and their final concentrations ranged from 4.0 to 0.031 µg/mL and from 1,280 to 5.0 µg/mL, respectively. Then, 100 µL of *E. faecalis* suspension (1 × 10^6^ CFU/mL) in double-concentrated BHI was mixed with 100 µL of CPC, AgNO_3_, or a CPC + AgNO_3_ solution on a 96-well microtiter plate. The untreated *E. faecalis* suspensions were used as the negative control group (NG), whereas the blank control group (BLK) contained only BHI. After anaerobic incubation at 37 °C for 24 h in the dark, the OD_600_ value was read using a micro-plate reader (Power Wave XS2, BioTek Instruments, VT, USA). The MIC was defined as the minimum concentration with a similar OD_600_ value as that of the BLK suspensions. Then, 10 µL of each of the suspensions whose concentration was ≥ MIC was added to BHI agar plates and cultured at 37 °C for another 24 h. The lowest concentration at which more than 99% of the original bacteria were killed was recorded as the MBC. All tests were repeated six times.

### Evaluation of the fractional inhibitory concentration index (FICI)

The combination effects of CPC + Ag^+^ were determined by calculating the FICI value as follows [2]: FICI_combined_ = FIC_CPC_ + FIC_Ag+_ = (MIC of CPC tested/MIC of CPC alone) + (MIC of AgNO_3_ tested/MIC of AgNO_3_ alone). The FICI was interpreted as follows: (1) a synergistic effect when the FICI ≤ 0.5; (2) an additive or indifferent effect when 0.5 < FICI ≤ 4; and (3) an antagonistic effect when the FICI > 4.

### Colony-forming unit (CFU) counting

The CFU counting method [18] was used to determine the antibacterial effects of CPC + Ag^+^ against planktonic *E. faecalis*. In brief, 1 mL of a *E. faecalis* suspension (2 × 10^5^ CFU/mL) in double-concentrated BHI broth was incubated with 1 mL of the tested solution at 37 °C in an anaerobic incubator for 24 h. The combinations with a FICI ≤ 0.5 were selected as the lowest synergetic concentration mixtures. The final concentrations of the test groups were 0.5 µg/mL CPC, 80 µg/mL AgNO_3_, 0.5 µg/mL CPC + 80 µg/mL AgNO_3_, 0.5 µg/mL CPC + 40 µg/mL AgNO_3_, 0.25 µg/mL CPC + 80 µg/mL AgNO_3_, and 0.25 µg/mL CPC + 40 µg/mL AgNO_3_. An untreated *E. faecalis* suspension was used as NG, and 2% CHX was used as the positive control. After performing serial 10-fold dilutions or not, 100 µL of each suspension was inoculated on a BHI agar plate for another 24 h. The *E. faecalis* CFU were then counted. The test was performed six times.

### Time-killing curve based on the CFU

According to our prior publication [34], the concentration of an *E. faecalis* suspension was adjusted to 2 × 10^6^ CFU/mL using double-concentrated BHI broth. Then, 1 mL of a *E. faecalis* suspension was incubated in the dark together with 1 mL of test solution at 37 °C in an anaerobic incubator. After 1, 3, 6, 9, and 24 h of incubation, a 10 µL test solution from each group, after being serially diluted 10-fold or not, was inoculated on a BHI agar plate under the same conditions for another 24 h. The *E. faecalis* CFU on the agar plate were then counted. The test was conducted six times.

### Dynamic growth curve

Referring to previous research [35], the concentration of an *E. faecalis* suspension was adjusted to 2 × 10^8^ CFU/mL using double-concentrated BHI broth before being mixed with 1 mL of the tested suspension and was then incubated at 37 °C in an anaerobic incubator with 5% CO_2_ and 1% O_2_ in the dark. The untreated *E. faecalis* suspension was designated as NG, whereas the BLK contained BHI only. The OD_600_ value of each group was measured using a micro-plate reader every 2 h within 10 h, and re-read at 20 and 24 h. The test was repeated six times.

### Cytotoxicity assays

The cytotoxicity was evaluated using a cell counting kit-8 (CCK-8, Dojindo Laboratories, Kumamato, Japan) according to a prior study [36]. A volume of 100 µL of α-minimum essential media (α-MEM) containing MC3T3-E1 cells (ATCC CRL-2594) was seeded on a 96-well plate (10^4^ cells per well) and 6 replicate wells were used for each group. After 24 h of incubation at 37 °C, the original media were replaced by 200 µL of culture medium (containing 10 µL of the test solutions) and further incubated. The culture medium was refreshed every 2 days. After incubation for 1, 3, and 7 days, the cells were washed twice with sterile phosphate-buffered saline (PBS). Then, 110 µL of a pre-mixed solution containing 100 µL of α-MEM and 10 µL of CCK-8 reagent was added to cells, and after 1 h of incubation in the dark, the absorbance of the cell suspensions of each group was measured at 450 nm (OD_450_) using a micro-plate reader.

### Anti-biofilm test on dentin slices

The method used in this experiment was based on previous studies [37, 38]. Dentin slices were made under the approval (2021–A46) of the Ethics Committee of School and Hospital of Stomatology, Wuhan University, and cut into 4 mm (width) × 4 mm (length) × 1 mm (thickness) pieces. After thoroughly ultrasound cleaning them using ddH_2_O, 5.25% NaClO, 17% ethylene diamine tetra-acetic acid (EDTA), and ddH_2_O again, in this sequence, for 4 min each, all dentin slices were autoclaved in ddH_2_O at 121 °C for 20 min. Then, each sterilized dentin slice was immersed in 1 mL of a *E. faecalis* suspension (1 × 10^8^ CFU/mL) and incubated in anaerobic conditions at 37 °C, and the original medium was replaced with fresh BHI every 48 h. After being cultured for 4 weeks, all dentin slices were gently washed three times with sterile PBS and randomly divided into 7 groups (n = 6).

To prepare the testing carboxymethyl cellulose gels, 0.05 g of carboxymethyl cellulose sodium (Aladdin Industrial Corp., Shanghai, China) was added to 2 mL of test solution. The BLK was composed of PBS carboxymethyl cellulose gel, and the 2% CHX carboxymethyl cellulose gel was used as the positive control. To simulate clinical intracanal medication, each of the dentin slices was embedded in a testing carboxymethyl cellulose gel for 7 days in the dark. After the 7-day treatment, each dentin slice was gently washed twice with sterile PBS and incubated on a vibration plate in 1 mL of sterile PBS for 1 min (20 s × 3 times). A volume of 10 µL of the solution was retrieved to inoculate a BHI agar plate, which was then incubated for another 24 h at 37 °C in anaerobic conditions to detect whether viable bacteria were still present on the dentin surface. Then, each dentin slice was soaked in 2 mL of fresh BHI to observe whether viable bacteria were still present in the dentinal tubes, and 200 µL suspensions were retrieved at 2, 4, 6, 8, 10, 12, and 24 h to measure their OD_600_ value using a microplate reader.

Additionally, one more slice from each group was observed using field emission scanning electron microscopy (FE-SEM)(Sigma, Zeiss, Germany) to observe the morphology of *E. faecalis* and its biofilms.

### Statistical analysis

All data in this study are presented as the mean ± standard deviation (SD). GraphPad Prism 8 (San Diego, CA, USA) was used for data analysis. For the CFU and cytotoxicity assays, a one-way analysis of variance (ANOVA) was conducted along with a *post-hoc* Dunnett t-test. For the time-killing curve, the dynamic growth curve, and the biofilm experiments, a two-way ANOVA was conducted. The statistical significance level was set at *p* < 0.05.

## Results

### MIC/MBC and the FICI values

The MIC/MBC and FICI results are shown in Table 1. After adding 0.5 µg/mL CPC, the MIC and MBC of AgNO_3_ sharply decreased to 20 and 40 µg/mL, respectively, whereas after adding 0.063 µg/mL CPC, only the MBC of AgNO_3_ decreased. Moreover, the FICI values of all groups were below 1.0, except when the dose of CPC used was 0.063 µg/mL.

**Table 1:** MIC/MBC and FICI of AgNO_3,_ CPC and AgNO_3_ with the addition of CPC

	MIC (µg/mL)	MBC (µg/mL)	FICI
AgNO_3_	160	640	/
CPC	1.0	4.0	/
0.5 µg/mL CPC + AgNO_3_	20	40	0.63
0.25 µg/mL CPC + AgNO_3_	40	80	0.5
0.125 µg/mL CPC + AgNO_3_	80	160	0.63
0.063 µg/mL CPC + AgNO_3_	160	320	1.06

/: ineligible to calculate

### CFU counting

**Fig. 1:**
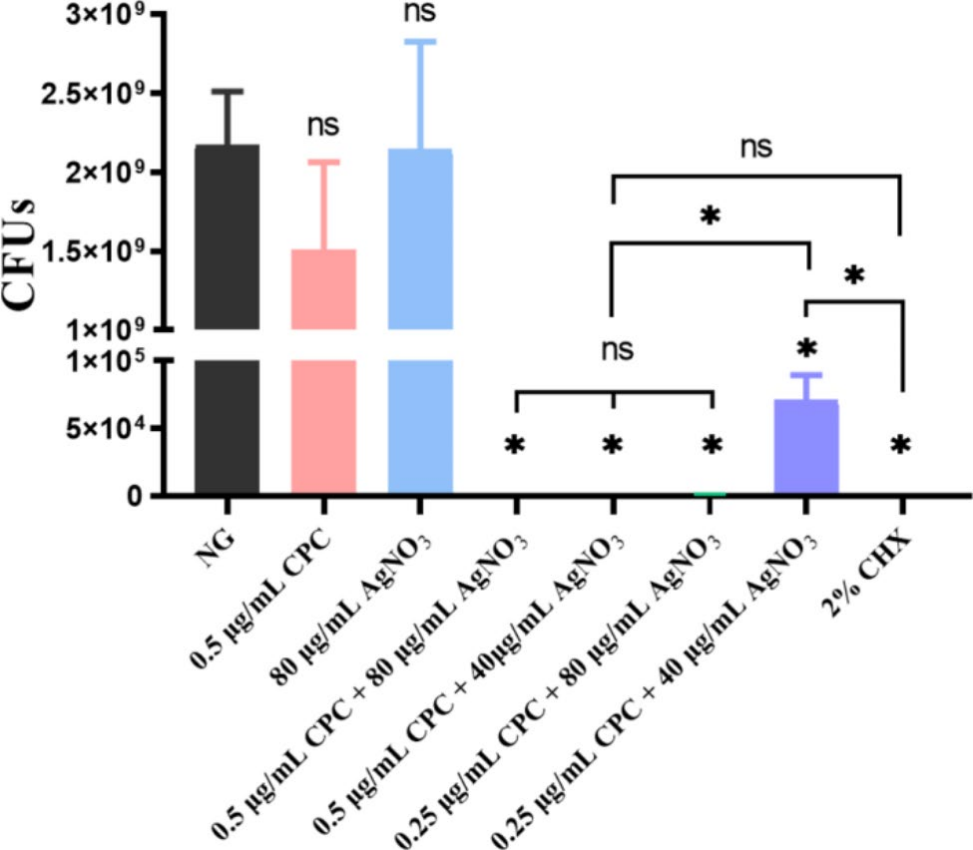
Antibacterial efficiencies of different concentrations of CPC + Ag^+^ against planktonic *E. faecalis* for 24 h. Compare the bacterial CFU counts among NG with tested groups (n = 6). NG: negative control group (*E. faecalis* without treatment). As compared with NG or inter-groups, ns: no significant difference, *: *p* < 0.05

All combination groups presented significantly lower CFU counts than NG or the groups in which each of the agents was used alone (*p* < 0.05; Fig. 1). When 0.5 µg/mL CPC was combined with 40 and 80 µg/mL AgNO_3_, no bacteria was found on the plate. The same results were obtained for the 2% CHX group.

### Time-killing curve

**Fig. 2:**
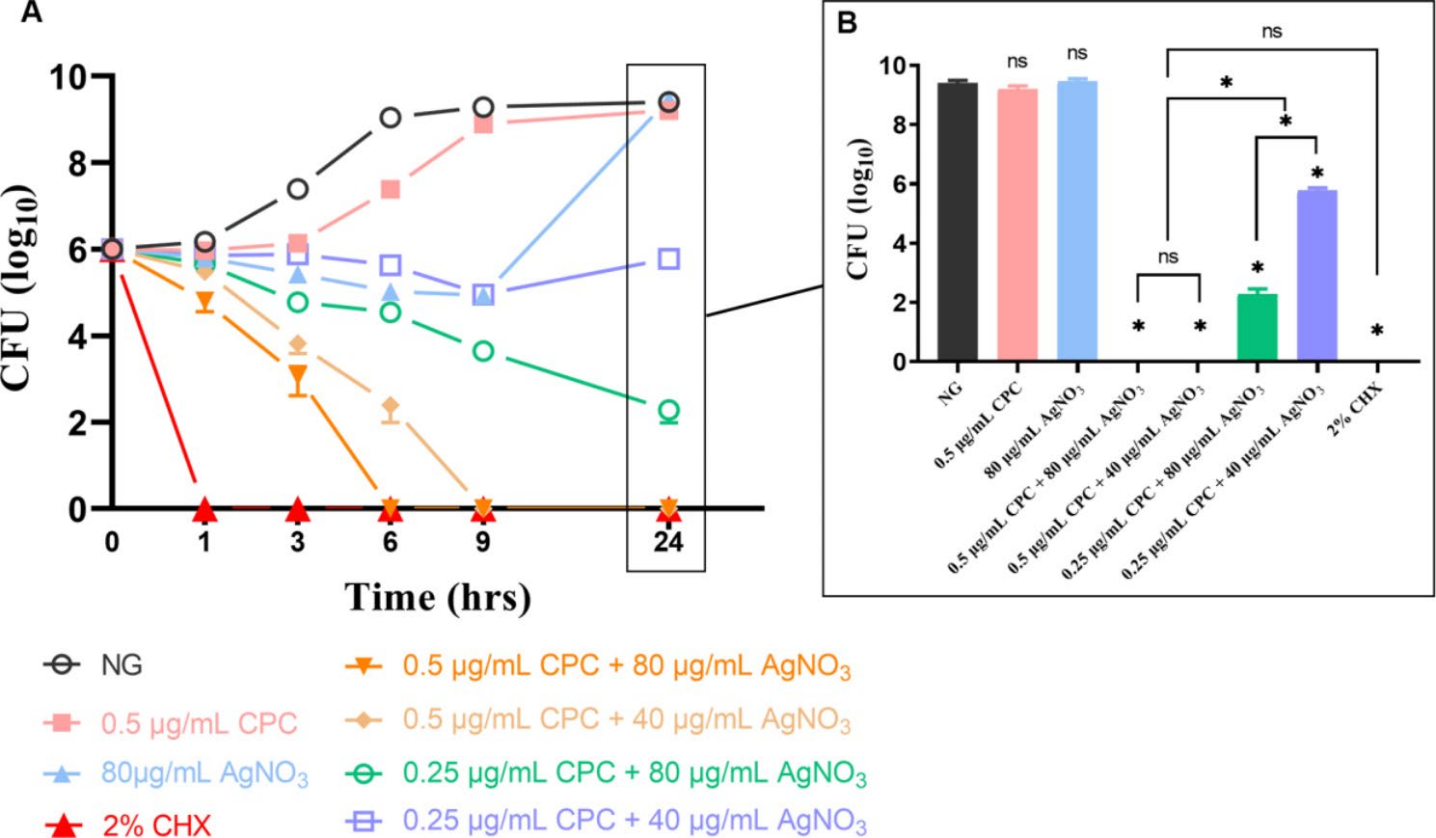
Time-killing curves of different concentrations of CPC + Ag^+^ against planktonic *E·faecalis* within 24 h. Values are expressed in log_10_ of CFU (n = 6). For the convenience, log_10_ is defined as 0 (CFU = 1) when no bacteria was founded on BHI agar plate. **A** time-killing curves within 24 h. **B** the CFU counting of each group at 24 h. NG: negative control group (*E. faecalis* without treatment). As compared with NG or inter-groups: ns: no significant difference, *: *p* < 0.05

Within 9 h, all the combination groups showed a dramatical or slight downward trend. During the 9 to 24 h period, only the 0.25 µg/mL CPC + 40 µg/mL AgNO_3_ group showed a growth trend, while the other combination groups showed a descending trend (Fig. 2A). Furthermore, the number of CFU counting after co-culture for 24 h (Fig. 2B) reflected the stable and significant antibacterial effect of CPC + Ag^+^.

### Dynamic growth curve

**Fig. 3:**
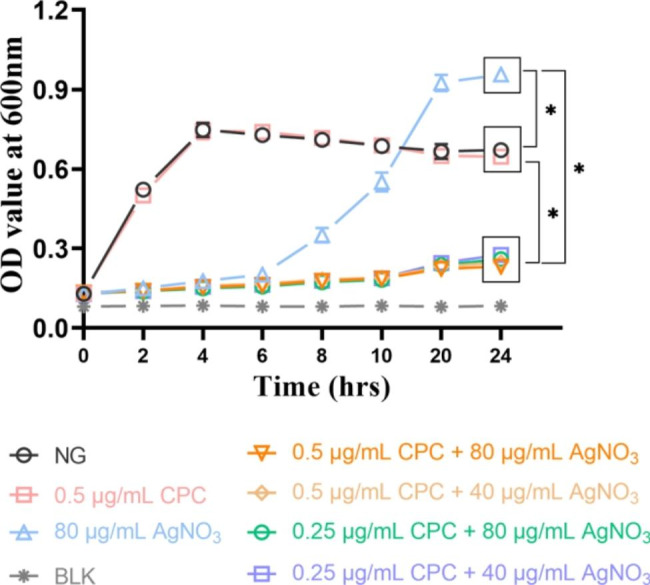
Dynamic growth curves of *E·faecalis* under different concentrations of CPC + Ag^+^ within 24 h. NG: negative control group (*E. faecalis* without treatment). BLK: blank control group (only BHI medium). *: *p* < 0.05 as compared inter-groups

The results indicate the inhibitory effect of CPC + Ag^+^ on *E. faecalis* for high initial bacterial density conditions (Fig. 3). The 0.5 µg/mL CPC group showed nearly the same results as NG within 24 h. However, the OD_600_ value of the 80 µg/mL AgNO_3_ group slightly increased within 6 h and significantly increased between 8 and 20 h. Comparatively, the OD_600_ value of all the combination groups did not change much within 10 h and only slightly increased between 10 and 24 h incubation (*p* < 0.05).

### Cytotoxicity test

**Fig. 4:**
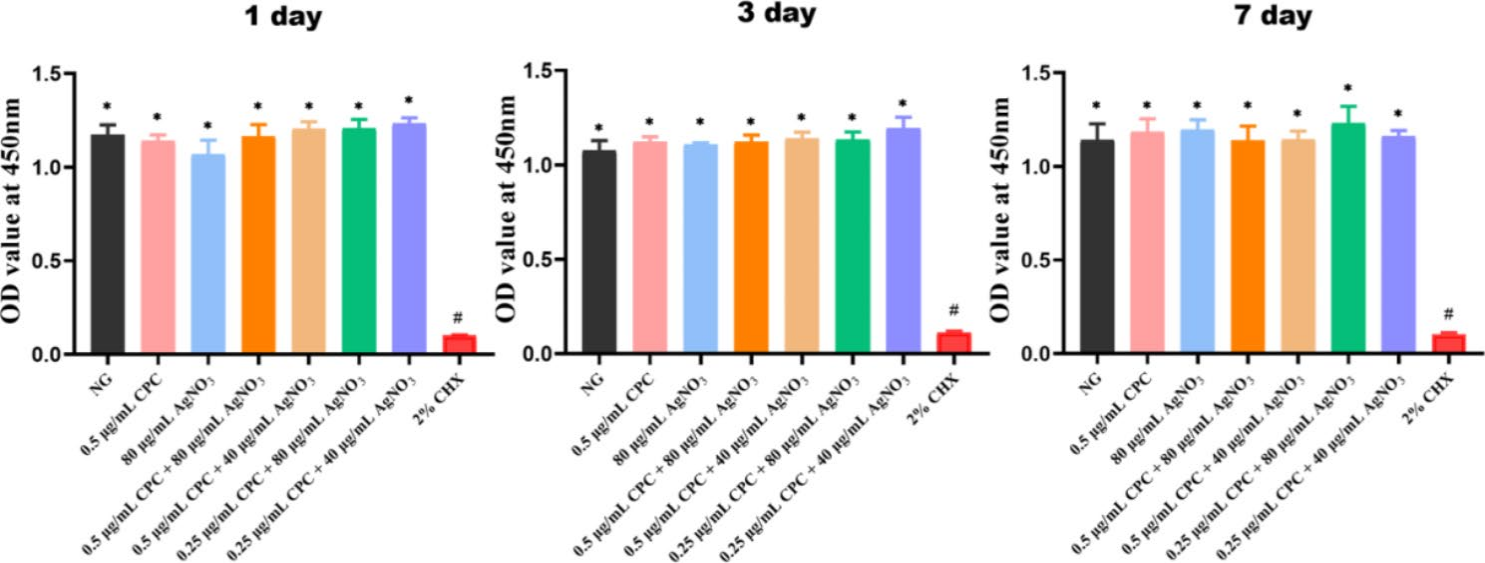
Cytotoxicity on MC3T3 -E1 cells using CCK-8 test result with culture time of 1 day, 3 days and 7 days. MC3T3 -E1 cells (1 × 10^4^ cells) exposed with 200 µL culture medium (containing 10 µL test solutions) (n = 6). NG: negative control group (MC3T3 -E1 cells without treatment). *: *p* < 0.05 as compared with 2% CHX, #: *p* < 0.05 as compared with other tested groups

Compared with the OD_450_ value of NG, those of all test groups showed no significant differences, whereas that of the CHX group significantly decreased, reflecting an inhibitory effect on cell proliferation (Fig. 4, *p* < 0.05).

### Anti-biofilm test on dentin slices

**Fig. 5:**
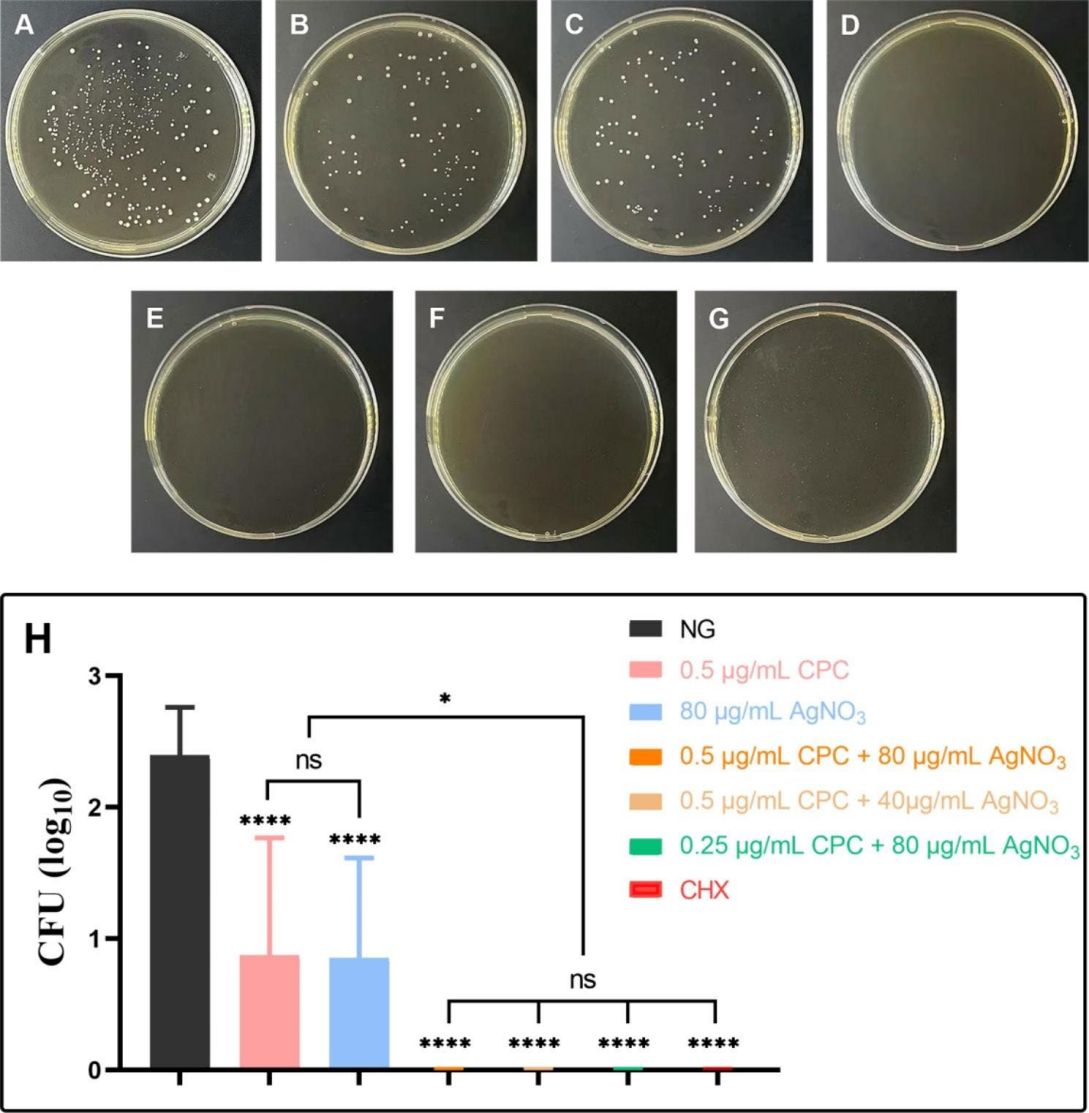
(**A-G**) Representative images of CFU of bacteria survived on the surface of dentine slices after treated. **A** NG, **B** 0.5 µg/mL CPC, **C** 80 µg/mL AgNO_3_, **D** 2% CHX; **E** 0.5 µg/mL CPC + 80 µg/mL AgNO_3_, **F** 0.5 µg/mL CPC + 40 µg/mL AgNO_3_, **G** 0.25 µg/mL CPC + 80 µg/mL AgNO_3_. **H** The CFU counting result of each group. NG: negative control group. As compared with NG or inter-groups: ns: no significant difference, *: *p* < 0.05, ****: *p* < 0.0001

**Fig. 6:**
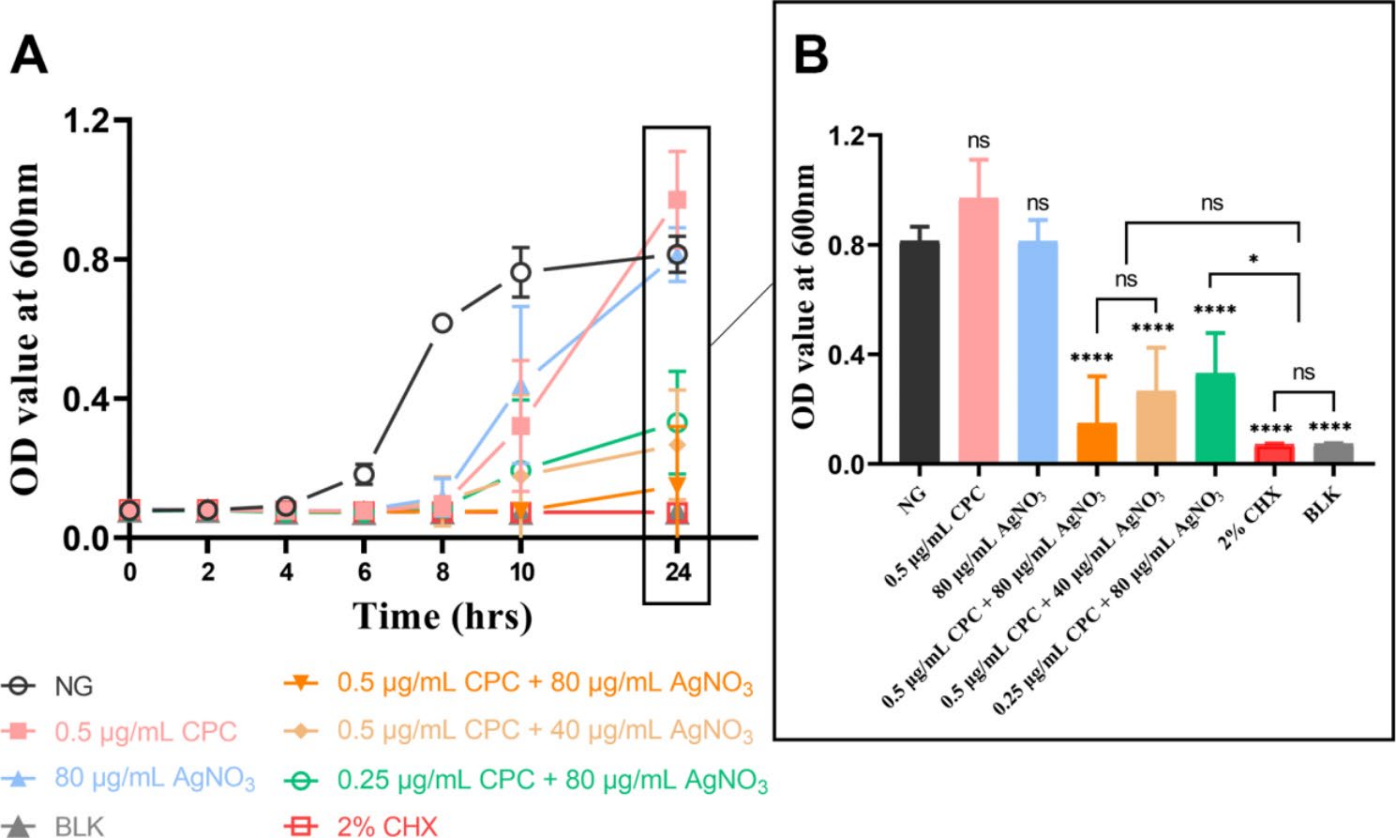
The dynamic curves of each group after the treated dentin slices soaking in fresh BHI. **A** The curves of OD_600_ value within 24 h after the direct soaking in fresh BHI media. **B** The OD_600_ value at 24 h. NG: negative control group. BLK: blank control group. As compared with NG or inter-groups: ns: no significant difference, *: *p* < 0.05, ****: *p* < 0.0001

**Fig. 7:**
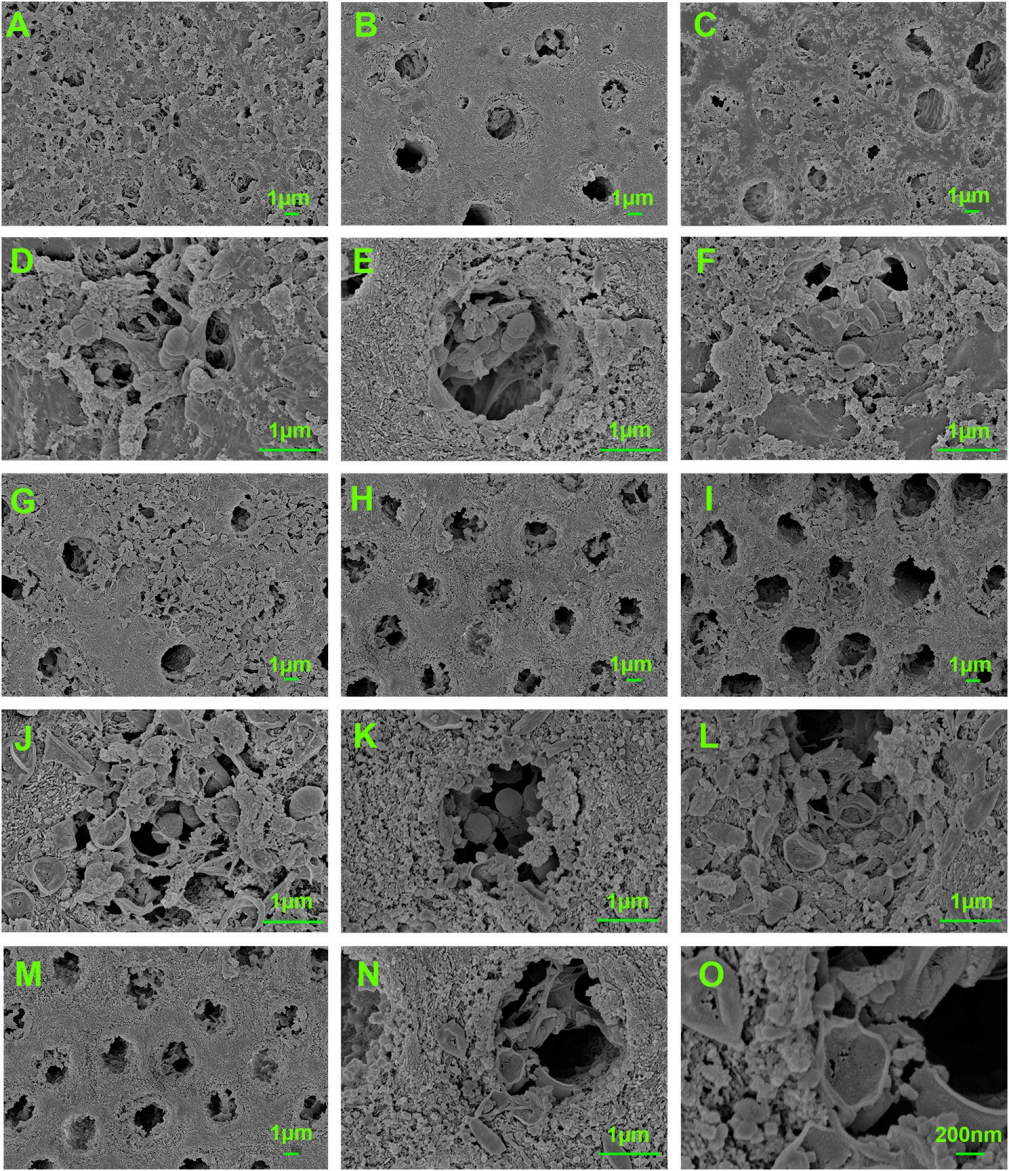
The FE-SEM images showed the differences in *E·faecalis* morphology and biofilms among (**A**, **D**) biofilms treated with PBS (**A** ×5000; **D** ×20,000); (**B**, **E**) biofilms treated with 0.5 µg/mL CPC only (**B** ×5000; **E** ×20,000); (**C**, **F**) biofilms treated with 80 µg/mL AgNO_3_ only (**C** ×5000; **F** ×20,000); (**G**, **J**) biofilms treated with 2% CHX only (**G** ×5000; **J** ×20,000); (**H**, **K**) biofilms treated with 0.25 µg/mL CPC + 80 µg/mL AgNO_3_ (**H** ×5000; **K** ×20,000); (**I**, **L**) biofilms treated with 0.5 µg/mL CPC + 40 µg/mL AgNO_3_ (**I **×5000; **L** ×20,000); (**M**, **N**, **O**) biofilms treated with 0.5 µg/mL CPC + 80 µg/mL AgNO_3_ (**M** ×5000; **N **×20,000; **O** ×50,000)

After a 7-day treatment, the CFU counting results (Fig. 5) revealed that almost no bacteria survived on the surface of the dentine slice after being treated with all combination groups and with the 2% CHX group. The dynamic curves result (Fig. 6) corresponds to the residual bacteria in the dentin tubules. The OD_600_ value of all combination groups showed significant difference compared with that of NG after 24 h incubation, and the OD_600_ value of 2% CHX was same as BLK and did not increased within 24 h. For combination groups, all of their OD_600_ values did not change much within 6 h and slightly increased after 8 h. Besides, the OD_600_ value of all combination groups showed no significant difference with BLK and 2% CHX after 24 h, except for the group of 0.25 µg/mL CPC + 80 µg/mL AgNO_3_. The FE-SEM images showed the differences in bacterial morphology and biofilms among groups. After treatment with the combination groups, the bacterial morphology changed dramatically, with most cells shrinking or breaking, and the mass of biofilms decreased (Fig. 7).

## Discussion

This study mainly focused on the co-antibacterial activity of CPC combined with Ag^+^ against *E. faecalis*. Results confirmed the synergistic antibacterial activity of a low-dose CPC + Ag^+^ mixture against planktonic *E. faecalis* and its antibacterial effect was concentration- and time-dependent. Though biofilm-resident bacteria are more resistant to antimicrobial agents than planktonic bacteria [39], the gel containing this mixture also showed antibacterial effectiveness on 4-week *E. faecalis* biofilms. According to the FE-SEM results, the residual biofilms and bacteria showed varying degrees of damage or dissolution, whereas the bacteria remained intact after treatment with 2% CHX. Furthermore, the cytotoxicity test indicated that low-dose CPC + Ag^+^ mixtures were more biocompatible than 2% CHX in the same conditions. These results indicated that low-dose CPC could improve the susceptibility of both planktonic and biofilm-resident *E. faecalis* to Ag^+^, and the combination of low-dose CPC + Ag^+^ showed enhanced antibacterial activity and good biocompatibility, which may have the potential to be developed into a novel root canal disinfectant.

Due to its biofilm lifestyle, *E. faecalis* has a remarkable capacity to infiltrate deeply into dentin tubules and survive an antibacterial environment even after thoroughly treatment [1, 39]. A biofilm consists of the cellular fraction and the extracellular polymeric substances (EPS). The latter accounts for most of the biofilm and is critical to protecting the embedded bacteria from antimicrobials and environmental threats [39, 40]. A high-dose antibacterial agent was usually required to inactivate the biofilm-resident bacteria; however, high-dose often leads to high cytotoxicity and some adverse side effects [41]. Fortunately, low-dose CPC + Ag^+^ seems to be both effective and biocompatible.

CPC is a cationic amphiphilic quaternary compound assembled by combining a positively charged pyridine, a hydrophilic headgroup, with a hexadecane chain, a lipophilic side chain [23, 42]. The positively charged pyridine head of CPC can replace the cations in the membrane; then, the hexadecane tail integrates into the lipid membrane and disorganizes it [43, 44]. CPC will cause membrane disintegration and cytoplasmic leakage at high doses, whereas at low doses, it merely impacts the cell by interfering with its osmoregulation and homeostasis [25]. Therefore, low-dose CPC may only play an auxiliary role in the antibacterial activity of the mixture. Meanwhile, recent studies have shown that *E. faecalis* could develop drug resistance to CHX, but not to CPC [26, 30]. Some other oral bacterial species, such as *Porphyromonas gingivalis* and *Streptococcus mutans* could be induced to CPC-resistance under the same condition [25, 45]. CPC might be a suitable disinfection agent for coping with *E. faecalis* infections.

The exact antibacterial mechanism of Ag^+^ is still unclear, although many extensive studies have been conducted on this topic. The probable molecular mechanism of its antibacterial action is related to the direct damage of the cell membrane, the extensive disruption of intracellular biomolecules, and the production of reactive oxygen species (ROS) [46, 47]. The initial damage of the cell membrane is crucial for the subsequent entry and accumulation of Ag^+^ in the cell, which inflicts additional damage on the cellular functions [10]. However, bacteria can become resistant to Ag^+^ via detoxification in the cytoplasm or by blocking Ag^+^ from entering the cell [46]. The changes in the membrane permeability and the binding of Ag^+^ to EPS both result in the inhibition of the entry of Ag^+^ into cells [15, 48]. Based on this information, we hypothesized that low concentrations of CPC could increase the permeability of cell membranes, making it easier for Ag^+^ to enter the bacteria and cause cell death, which was demonstrated as an increased susceptibility of *E. faecalis* to Ag^+^. Despite these findings in this study, the mechanism behind the synergistic enhanced antibacterial activity between low-dose CPC + Ag^+^ still needs to be further investigated. Besides, it is also necessary to find suitable carrier biomaterials for loading and releasing CPC and Ag^+^ for an optimal antibacterial effect in root canals.

## Conclusions

This study evaluated the effectiveness and feasibility of the combination of low-dose CPC and Ag^+^. The sensitivity of both planktonic and biofilm *E. faecalis* against Ag^+^ was significantly enhanced after the addition of low-dose CPC. Conversely, the cytotoxicity of low-dose CPC was much lower than that of 2% CHX. Therefore, the combination of low-dose CPC + Ag^+^ may be used as a novel and potent antibacterial mixture against *E. faecalis* with low toxicity for root canal disinfection or other related medical applications.

## Data Availability

All data generated or analyzed during this study are included in this published article.
